# Risk factors for the development of bronchiectasis in patients with asthma

**DOI:** 10.1038/s41598-021-02332-w

**Published:** 2021-11-24

**Authors:** Donghai Ma, María-Jesús Cruz, Iñigo Ojanguren, Christian Romero-Mesones, Diego Varona-Porres, Xavier Munoz

**Affiliations:** 1 Servicio de Neumología, Hospital Universitari Vall d’Hebron, Barcelona (HUVH) Institut de Recerca Vall d’Hebron (VHIR), Universitat Autónoma de Barcelona, Passeig Vall d’Hebron, 119, 08035 Barcelona, Spain; 2 Centro de Investigación en Red de Enfermedades Respiratorias (CIBERES), Instituto de Salud Carlos III (ISCIII), Madrid, Spain; 3 Departamento de Fisiología, Universitat Autónoma de Barcelona, Barcelona, Spain; 4 Servicio de Radiología. Hospital Universitari Vall d’Hebron, Barcelona, Spain

**Keywords:** Asthma, Medical research

## Abstract

Though asthma and bronchiectasis are two different diseases, their coexistence has been demonstrated in many patients. The aim of the present study is to compare the characteristics of asthmatic patients with and without bronchiectasis and to assess risk factors for the development of this condition. Two hundred and twenty-four moderate-severe asthmatic patients were included. The severity of bronchiectasis was assessed by Reiff and FACED parameters. Logistic regression was used to identify independent factors associated with bronchiectasis. Bronchiectasis was identified in 78 asthma patients. In severe asthma patients, its prevalence was 56.9%. Bronchiectasis was defined as mild in81% of patients using modified Reiff criteria and in 74% using FACED criteria. Asthmatic patients with bronchiectasis had decreasing FEV1, FVC and FEV1/FVC (*p* = 0.002, 0.005 and 0.014 respectively), presented more frequent asthma exacerbations (*p* < 0.001) and worse asthma control (ACT 21 vs 16pts, *p* < 0.001). Factors independently associated with bronchiectasis were older age (42–65 years: OR, 3.99; 95% CI 1.60 to 9.95, *P* = 0.003; ≥ 65 years: OR, 2.91; 95% CI 1.06 to 8.04, *P* = 0.039), severe asthma grade (OR, 8.91; 95% CI 3.69 to 21.49; *P* < 0.001) and frequency of asthma exacerbations (OR, 4.43; 95% CI 1.78 to 11.05; *P* < 0.001). In patients with severe asthma, age of asthma onset (OR, 1.02; 95% CI 1.01 to 1.04; *P* = 0.015) and asthma exacerbations (OR, 4.88; 95% CI 1.98 to 12.03; *P* = 0.001) were independently associated with the development of bronchiectasis. The prevalence of bronchiectasis in severe asthmatic patients is high. Age of asthma onset and exacerbations were independent factors associated with the occurrence of bronchiectasis.

## Introduction

Asthma is a disease characterized by chronic airway inflammation. It is estimated that asthma affects about 339 million people worldwide, and its prevalence is increasing rapidly^1,2^. Asthma symptoms may be triggered or worsened by many factors: biological factors such as viral or microorganism infections, physiological factors such as exercise and cold air, and environmental triggers such as exposure to cigarettes, pollen, and diesel exhaust particles^3–5^.

For a long time, bronchiectasis (BQ) was considered an orphan disease^6,7^. The Spanish guidelines on the Evaluation and Diagnosis of Bronchiectasis in Adults^8^ define BQ as a heterogeneous chronic airway disease with bronchial dilatation, accompanied by clinical symptoms such as cough, chronic expectoration and recurrent lung infections. Bronchiectasis is now reported to be the third most frequent airway chronic disease, just behind asthma and chronic obstructive pulmonary disease (COPD)^1^.

The coexistence of asthma and BQ has been reported previously. Indeed, the prevalence of BQ in asthmatic patients ranges from 3% in general asthmatic patients, without specification of asthma severity, to 47–67.5% in patients with severe asthma^9–11^. In some studies of BQ the prevalence of asthma has also been reported^12,13^. It seems that there are some underlying correlations between asthma and bronchiectasis; however, the evolution of BQ and its pathophysiology require long term follow-up studies^14^. Little is known about the impact of BQ on asthma, its clinical manifestation, comorbidities and disease management. The presence of BQ may also trigger asthma exacerbations and may challenge the management of the disease.

The aim of the present study is to compare the characteristics of asthmatic patients with and without BQ and to assess risk factors for the development of BQ, using data consecutively recorded at a specific asthma unit over a one–year period.

## Materials and methods

### Study population and design

**Figure 1:**
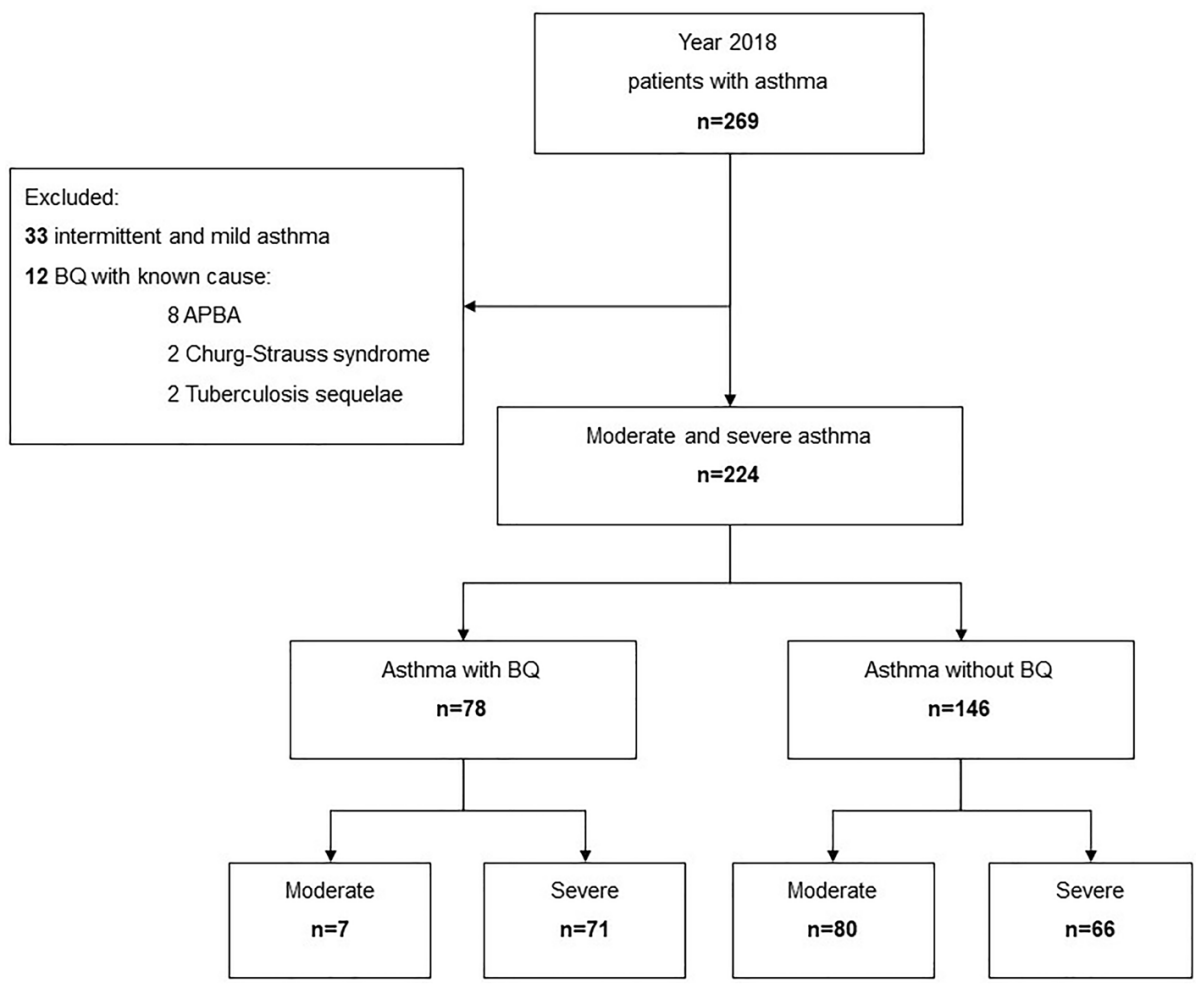
Flow chart of the study population. ABPA: allergic bronchopulmonary aspergillosis; BQ: non-fibrosis bronchiectasis.

We performed an ambispective study of 269 asthma patients seen at our specialized asthma unit in 2018. Of these, 33 with intermittent and mild asthma, and twelve with known etiology of BQ (eight Churg-Strauss syndrome, two allergic bronchopulmonary aspergillosis, and two sequelae post-tuberculosis) were excluded. The remaining 224 patients with moderate and severe asthma were finally included in the study (Fig. 1).

Clinical histories of all patients included and data including anthropometric, smoking habits, comorbidities, asthma history, asthma grade, atopy status and exacerbations were retrospectively reviewed. Variables associated with BQ and microorganism colonization in sputum culture, when they were available, were also reviewed and recorded. Patients were examined and asked about their present medication, and the Asthma Control Test (ACT)^15^ was administered. The records were checked to establish whether spirometry had been performed during the previous year, or a High-Resolution Computed Tomography (HRCT) during the last three years. If not, or if no data were available, these tests were administered. The study was approved by our hospital’s Ethics Committee (PR(AG)50/2019). All patients provided written informed consent to participate in the study.

### Asthma diagnosis and severity

The diagnosis of asthma was made according to GINA^16^ guidelines and based on clinical symptoms plus one or more complementary tests. All patients had shown reversible airway obstruction on bronchodilator testing, or a positive methacholine challenge test, or variability > 20% in the peak flow recording. Moderate and severe asthma were defined based on the GINA guidelines, which corresponded to patients taking steps 3, 4, and 5 of medication with at least 200 μg of inhaled corticosteroids (budesonide or equivalent doses) plus a long-acting beta-2 agonist^16^.

### Pulmonary function test

Spirometry was performed using a MasterLab instrument (MasterLab, Jaeger, Germany), according to European Respiratory Society (ERS) and American Thoracic Society (ATS) guidelines^17^. The reference values used were those proposed by the ERS^18^.

### Bronchiectasis diagnosis and severity

Bronchiectasis was identified in accordance with the Spanish Society of Pulmonology and Thoracic Surgery (SEPAR) recommendations, by comparing the internal bronchial lumen diameter with the adjacent artery calibre in HRCT images^8^. Morphological characteristics of BQ, including bronchodilation, bronchial wall thickening, dilatation type and lobe extension were reviewed and assessed according to the modified Reiff score^19^ in six lobes (18 points in total): 1–6 mild, 7–12 moderate, and ≥ 13 severe.

The FACED parameter^20^, which incorporates variables such as FEV1, age, pseudomonas colonization, lobe extension and dyspnea, was used for the clinical estimation of patients' status..

HRCT was performed with 1 mm cuts at 10 mm intervals in maximum inspiration. All the imaging features of BQ were interpreted by two pulmonologists working independently. For controversial images, an expert radiologist was consulted and the final decision was made. The degree of the inter-observer agreement was assessed by the Kappa statistic^21^.

### Atopy and smoking status

Patients were considered atopic if they had at least one positive prick test for any of the common environmental allergens. Non-smokers were patients who had never smoked, and ex-smokers were those who had not smoked for at least six months. The number of pack-years was calculated in all cases.

### Definition of exacerbations

Episodes of asthma exacerbations during the previous year were recorded. Asthma exacerbations were defined according to GINA guidelines^16^ which specify asthma attacks or acute asthma exacerbation as episodes of progressive increases in shortness of breath, cough, wheezing, or chest tightness, or some combination of these symptoms, accompanied by reductions in expiratory airflow.

### Statistical analysis

Categorical variables were presented as frequencies and percentages. Statistical differences were analysed using the Chi-square test (or Fisher's exact test when appropriate). Continuous variables were presented as means and standard deviations (SD), or as medians with interquartile ranges (IQR) when data were not normally distributed. The Shapiro–Wilk test was used to analyse the distribution of variables. A logistic regression model was used to determine the factors that were independently associated with the outcome, in this case, the presence of BQ. Variables that presented statistically significant differences (*p* < 0.05) in the bivariate analysis or were considered to be of clinical interest (such as gender) were included as independent variables in the first step. Variables associated with disease management and asthma control test score were not included. A forward stepwise technique (Wald test; removal threshold, *p* > 0.10) was used to perform this analysis. ORs and 95% CIs were calculated for independent variables. Tolerance and the Variance Inflation Factor (VIF) were examined to avoid multicollinearity among variables. Statistical significance was defined as a two-tailed *p* ≤ 0.05. The statistical analyses were performed using SPSS (version 25, Chicago, IL).

### Ethics approval and consent to participate

The study was approved by the hospital Vall d'Hebron Ethics Committee (Reference number: PR(AG)50/2019). All patients provided written informed consent prior to participating. All methods were performed in accordance with the relevant guidelines and regulations.

### Informed consent

All patients provided written informed consent to participate in the study.

## Results

The socio-demographic characteristics of patients with and without BQ are shown in Table 1. Asthma patients with BQ were older than patients without BQ (56.2 vs. 49.9 years, *p* = 0.005). Bronchiectasis was more frequent in middle-aged subjects (42–65 years) (62.8% vs 44.5%, *p* < 0.05), but this trend was reversed in the younger adult group (< 42 years) (11.5% vs 30.8%, *p* < 0.05). Furthermore, subjects with BQ had more severe asthma (91% vs 45.2%, *p* < 0.001) and presented a higher number of exacerbations (median 1 vs 0 episodes, *p* < 0.001). Significantly lower FVC% pred, FEV1%pred and the ratio of FEV1/FVC were observed in asthma patients with BQ than in those without (89.4% vs 82.8%, *p* = 0.005; 81.0% vs 73.2%, *p* = 0.002; 72.7 vs 69.5, *p* = 0.014 respectively). Subjects with BQ presented more sinusitis (10.3% vs 2.1%, *p* = 0.018) and nasal polyps (28.2% vs 13.0%, *p* = 0.005) than those without. Subjects without BQ seemed to be more atopic than those with BQ (63.0% vs 43.6%, *p* = 0.005). No significant differences were observed in the remaining comorbidities.

**Table 1:** Socio-demographic characteristics and clinical data of the study population.

	Moderate and severe asthma		*P*	Severe asthma		*P*
	Without BQ (146)	with BQ (78)		Without BQ (66)	with BQ (71)	
**Sociodemographic data**						
Gender (female)	94 (64.4%)	46 (59.0%)	0.43	37 (56.1%)	43 (60.6%)	0.59
Age (years)	49.9 (17.2)	56.2 (13.1)	**0.005**	49.7 (17.0)	56.2 (13.5)	**0.014**
Age group*			**0.004**			**0.033**
A1, < 42 yrs	45 (30.8%)	9 (11.5%)	** < 0.001**^†^	20 (30.3%)	9 (12.7%)	**0.009**^†^
A2, 42–65 yrs	65 (44.5%)	49 (62.8%)	0.364^††^	29 (43.9%)	43 (60.6%)	0.078^††^
A3, ≥ 65 yrs	36 (24.7%)	20 (25.6%)	0.023^¶^	17 (25.8%)	19 (26.8%)	0.491
Race (Caucasian)	141 (96.6%)	72 (92.3%)	0.20	63 (95.5%)	65 (91.5%)	0.50
BMI (kg/m^2^)	27.1 (23.6,30.7)	27.1 (24.4, 29.1)	0.77	27.9 (24.7, 30.7)	27.3 (24.6, 29.1)	0.35
Smoking status			0.24			0.42
Non-smoker	100 (68.5%)	46 (59.0%)		43 (65.2%)	42 (59.2%)	
Current-smoker	8 (5.5%)	3 (3.8%)		4 (6.1%)	2 (2.8%)	
Ex-smoker	38 (26.0%)	29 (37.2%)		19 (28.8%)	27 (38.0%)	
Packs-year	0.0 (0.0, 3.0)	0.0 (0.0, 15.0)	0.052	0.0 (0.0, 4.0)	0.0 (0.0, 15.0)	0.30
**Clinical data of asthma & lung function**						
Atopic asthma	92 (63.0%)	34 (43.6%)	**0.005**	42 (63.6%)	30 (42.3%)	**0.012**
Asthma grade			< **0.001**			NA
Moderate	80 (54.8%)	7 (9.0%)				
Severe	66 (45.2%)	71 (91.0%)				
Years of asthma	17.8 (9.4, 31.8)	19.4 (9.5, 37.1)	0.27	20.2 (13.3, 40.1)	20.4 (9.6, 37.3)	0.57
Age of asthma onset	26.0 (6.0, 45.1)	33.7 (16.0, 45.9)	0.20	20.5 (5.5, 37.6)	33.8 (15.0, 46.0)	**0.024**
CAO (yes)	49 (33.6%)	22 (28.2%)	0.41	25 (37.9%)	21 (29.6%)	0.30
ACT (pts)	21 (18, 24)	16 (13, 21)	**< 0.001**	20.0 (17.0, 23.0)	16.0 (13.0, 21.0)	**0.003**
Exacerbation	0.0 (0.0, 1.0)	1.0 (0.0, 3.0)	** < 0.001**	0.0 (0.0, 2.0)	2.0 (0.0, 4.0)	**< 0.001**
≥ 3 courses	8 (5.5%)	27 (34.6%)	**< 0.001**	8 (12.1%)	27 (38.0%)	** < 0.001**
FVC% pred	89.4 (15.9)	82.8 (17.7)	**0.005**	86.8 (16.9)	81.8 (17.7)	0.096
FEV1% pred	81.0 (17.1)	73.2 (19.6)	**0.002**	75.8 (17.1)	72.2 (19.4)	0.25
FEV1/FVC	72.7 (9.0)	69.5 (9.1)	**0.014**	70.2 (8.5)	69.5 (9.2)	0.62
FEV1/FVC ≥ 70	86 (58.9%)	42 (53.8%)	0.47	32 (48.5%)	34 (47.9%)	0.94
**Comorbidity**						
NSAIDs allergy	25 (17.1%)	13 (16.7%)	0.93	19 (28.8%)	11 (15.5%)	0.060
Rhinitis	50 (34.2%)	22 (28.2%)	0.36	21 (31.8%)	21 (29.6%)	0.78
Sinusitis	3 (2.1%)	8 (10.3%)	**0.018**	1 (1.5%)	8 (11.3%)	**0.034**
Nasal polyps	19 (13.0%)	22 (28.2%)	**0.005**	11 (16.7%)	20 (28.2%)	0.11
Obesity	46 (31.5%)	18 (23.1%)	0.18	23 (34.8%)	16 (22.5%)	0.11
WRA	25 (17.1%)	14 (17.9%)	0.88	10 (15.2%)	12 (16.9%)	0.78

Continuous variables expressed as mean (SD) or median (p25, p75); categorical data expressed as percentage n (%); *Bonferroni adjust method was used to perform comparison between age subgroup, significant p level at 0.017; †, A1 vs A2; ††, A2 vs A3; ¶, A1 vs A3). BMI: body mass index; CAO: childhood asthma onset (18 yrs cut-off); ACT: asthma control test; BQ: non-cystic fibrosis bronchiectasis; WRA, work-related asthma. Significant p values in bold font.

With regard to disease management (Table 2), subjects with BQ were more corticosteroid-dependent (34.6% vs 3.4%, *p* < 0.001) and had also taken more azithromycin (34.6% vs 6.8%, *p* < 0.001). In addition, the consumption of long-acting muscarinic receptor antagonists (LAMA) and anti-leukotriene was significantly higher in asthma patients with BQ than in those without (69.2% vs. 26.7%, *p* < 0.001 and 65.4% vs. 39.0%, *p* < 0.001 respectively).

**Table 2:** Comorbidities and disease management of the study population.

	Moderate and severe asthma		*P*	Severe asthma		*P*
	without BQ (146)	with BQ (78)		without BQ (66)	with BQ (71)	
**Comorbidity**						
NSAIDs allergy	25 (17.1%)	13 (16.7%)	0.93	19 (28.8%)	11 (15.5%)	0.060
Rhinitis	50 (34.2%)	22 (28.2%)	0.36	21 (31.8%)	21 (29.6%)	0.78
Sinusitis	3 (2.1%)	8 (10.3%)	**0.018**	1 (1.5%)	8 (11.3%)	**0.034**
Nasal polyps	19 (13.0%)	22 (28.2%)	**0.005**	11 (16.7%)	20 (28.2%)	0.11
Obesity	46 (31.5%)	18 (23.1%)	0.18	23 (34.8%)	16 (22.5%)	0.11
WRA	25 (17.1%)	14 (17.9%)	0.88	10 (15.2%)	12 (16.9%)	0.78
**Disease management**						
Cortisone-dependent	5 (3.4%)	27 (34.6%)	**< 0.001**	4 (6.1%)	27 (38.0%)	**< 0.001**
Budesonide (μg/d)	1040 (640,1600)	1280 (640,1600)	0.37	1440 (800,1600)	1400 (640,1600)	0.29
ICS dose category			0.14			0.61
Low	30 (20.5%)	10 (12.8%)		5 (7.6%)	9 (12.7%)	
Med	42 (28.8%)	18 (23.1%)		13 (19.7%)	14 (19.7%)	
High	74 (50.7%)	50 (64.1%)		48 (72.7%)	48 (67.6%)	
LAMA	39 (26.7%)	54 (69.2%)	**< 0.001**	29 (43.9%)	53 (74.6%)	** < 0.001**
Azithromycin	10 (6.8%)	26 (33.3%)	** < 0.001**	8 (12.1%)	26 (36.6%)	** < 0.001**
Omalizumab	18 (12.3%)	15 (19.2%)	0.16	18 (27.3%)	15 (21.1%)	0.40
Mepolizumab	4 (2.7%)	5 (6.4%)	0.28	4 (6.1%)	5 (7.0%)	1.00
Anti-leukotriene	57 (39.0%)	51 (65.4%)	**< 0.001**	39 (59.1%)	48 (67.6%)	0.30
Theophylline	2 (1.4%)	1 (1.3%)	1.00	2 (3.0%)	1 (1.4%)	0.61

Continuous variables expressed as mean (SD) or median (p25, p75); categorical data expressed as percentage n (%); BQ: non-cystic fibrosis bronchiectasis; NSAIDs, allergy to nonsteroidal anti-inflammatory drugs; LAMA: long-acting muscarinic receptor antagonists; ICS: Inhaled Corticosteroid. Significant p values in bold font.

The prevalence of BQ in severe asthma patients was 56.9% (Fig. 1). A sub-analysis limited to these subjects with severe asthma found similar differences in age, ACT, asthma exacerbation, atopy, sinusitis, cortisone dependency and asthma medication (LAMA and azithromycin). However, the differences in lung function were no longer statistically significant. Furthermore, patients with BQ had a later asthma onset than patients without (median 20.5 years’ vs 33.8 years, *p* = 0.024) (Tables 1 and 2).

**Figure 2:**
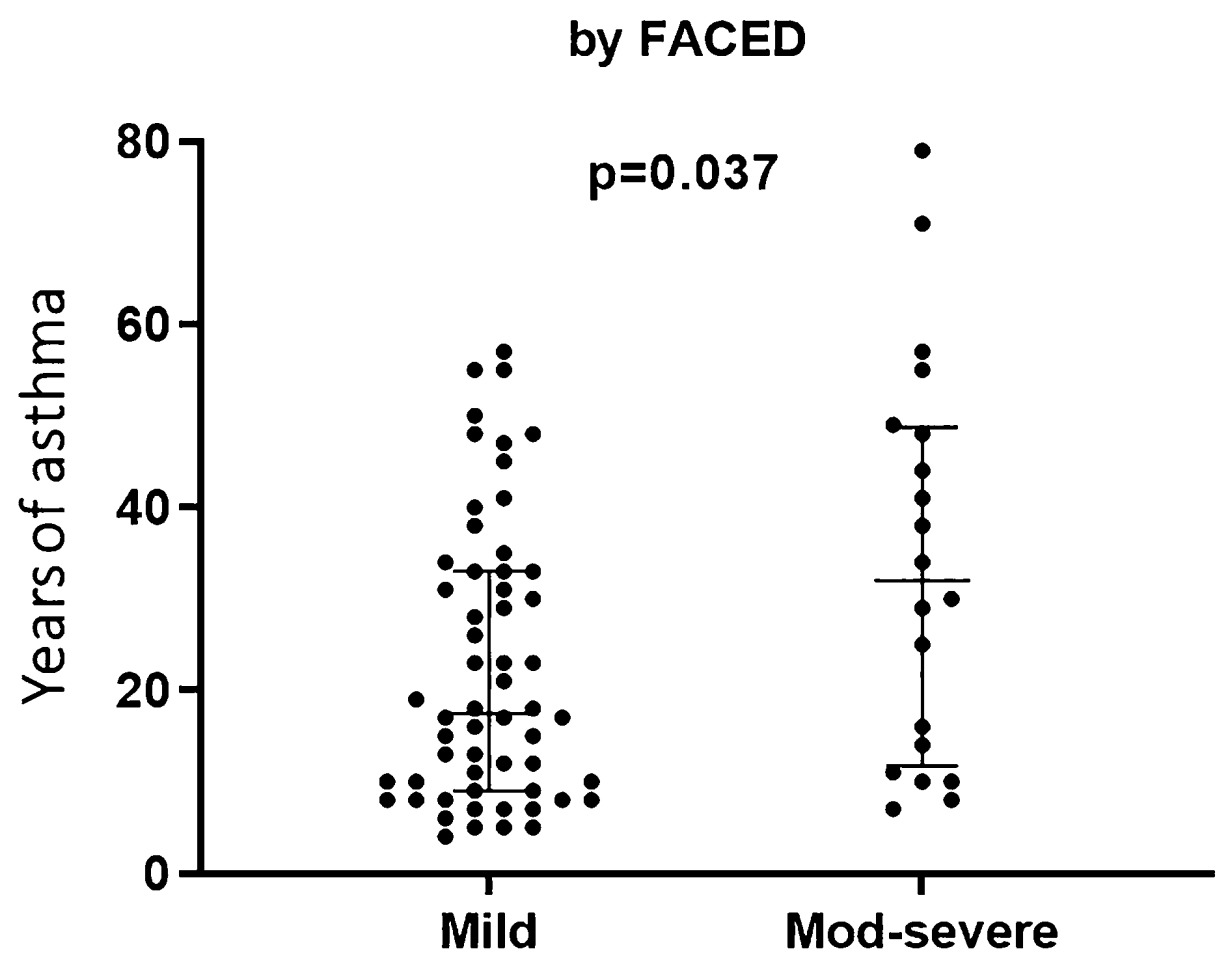
Asthma-year according to bronchiectasis severity assessed by FACED. Data expressed as median (IQR). Years of asthma 17.2 (9.1, 32.5) in mild vs 31.7 (12.0, 48.3) in moderate and severe bronchiectasis, *p* = 0.037.

Of the 78 patients with BQ, 81% were classified as mild according to modified Reiff criteria, and 74% according to FACED criteria (Table 3). Patients with mild BQ (according to FACED) had fewer years of asthma evolution than those with moderate and severe BQ (17.2 yrs vs 31.7 yrs, *p* = 0.037) (Fig. 2). A significant correlation between the FACED score and dyspnea (r = 0.66, *p* = 0.0001) was observed. Seventy-five patients (96%) had widespread bronchial dilatation, with all six lobes involved. Bronchial wall thickening was moderate in 68% of patients and mild in 27%. The predominant dilatation type was cylindrical (82%). Kappa values (and overall agreement %) for lobe extension, bronchial dilatation, bronchial wall thickening, and dilatation type were 0.83 (98.7%), 0.75 (88.5%), 0.70 (89.7%), and 0.79 (94.9%) respectively.

**Table 3:** Assessment of bronchiectasis by radiological and clinical parameters.

Comprehensive parameter		Individual parameter	
FACED		Lobe extension with lingula included	
		4 lobes involved	3 (4%)
		6 lobes involved	75 (96%)
Mild	58 (74%)	Bronchial wall thickening* (predominant)	
Moderate	18 (23%)	Mild	21 (27%)
Severe	2 (3%)	Moderate	53 (68%)
Modified Reiff		Severe	4 (5%)
		Dilatation type (predominant)	
Mild	63 (81%)	Cylindrical	64 (82%)
Moderate	13 (17%)	Varicose	12 (15%)
Severe	2 (3%)	Cystic	2 (3%)

Left part: systemic parameters. Right part: individual parameters. * Bronchial wall thickening by comparing with the adjacent artery diameter: mild < 0.5 A; moderate (0.5–1) A; severe > 1 A. Modified Reiff score: 1–6 pts mild, 7–12 pts moderate and ≥ 13 pts severe. FACED score: 0–2 pts mild, 3–4 pts moderate and 5–7 pts severe bronchiectasis.

Potentially pathogenic bacteria were identified in 16 subjects (21%) with BQ, including P. aeruginosa (five cases), H. influenzae (three), S. pneumonia (three), S. aureus (two), K. pneumonia (one), M. catarrhalis (one) and P. putida (one).

**Figure 3:**
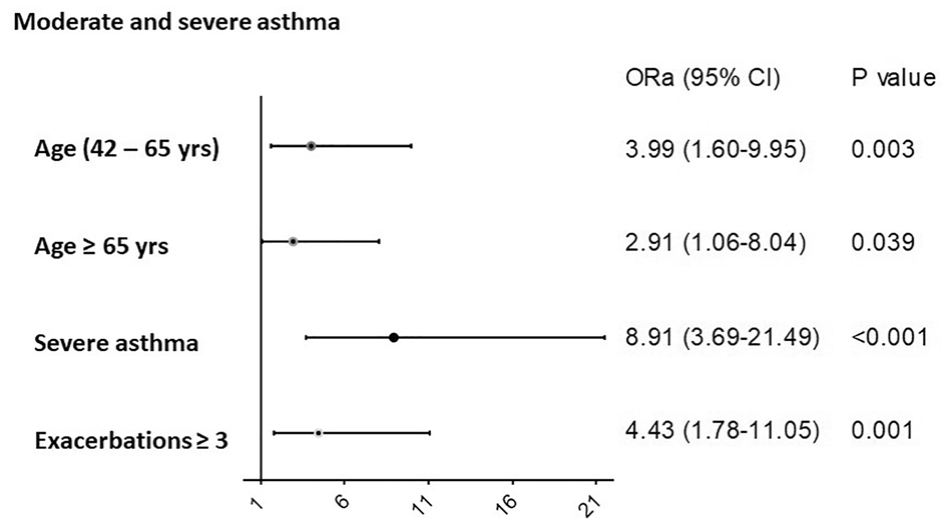
Factors associated with bronchiectasis in all subjects (moderate and severe asthma). ORa: adjusted odds ratio. CI: confidence interval. Model: χ^2^ = 73.64, *p* < 0.001. Model adjusted by gender, atopy, sinusitis, nasal polyps, FVC% and FEV1%. Logistic regression was used to perform the analysis.

**Figure 4:**
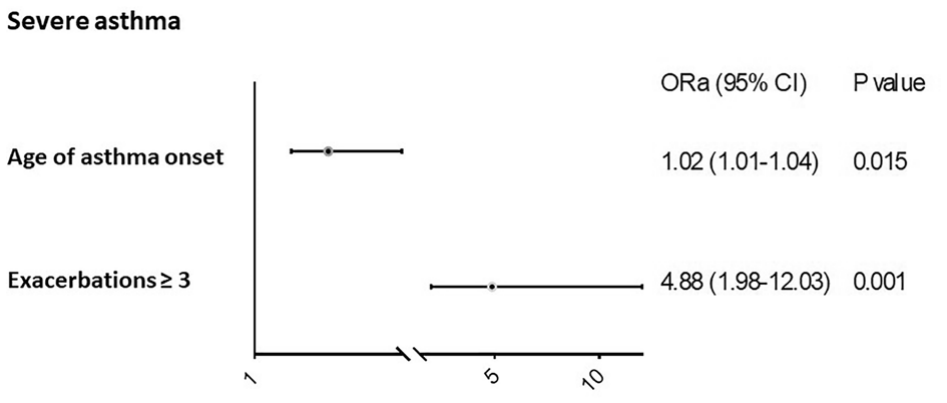
Factors associated with bronchiectasis in severe asthma patients. ORa: adjusted odds ratio. CI: confidence interval. Model: χ^2^ = 18.82, *p* < 0.001. Model adjusted for gender, age group, atopy and sinusitis. Logistic regression was used to perform the analysis.

The odds ratios and 95% confidence intervals of variables related to BQ in all asthma patients and in severe asthma patients are shown in Table 4 and in Figs. 3 and 4 respectively. In the entire group, age, severe asthma grade and asthma exacerbations (≥ 3) were independently associated with the presence of BQ, and in the severe asthma group, age of asthma onset and ≥ 3 exacerbations were independently associated with this outcome.

**Table 4:** Univariate and multivariate regression analysis of appearance of bronchiectasis in the study population.

Variables	Univariate			Multivariate		
	OR	95% CI	*p*	OR	95% CI	*p*
**Moderate and severe asthma patients**						
Age < 42 yrs			0.002			0.012
42–65 yrs	3.77	1.68–8.44	0.001	3.99	1.60–9.95	0.003
≥ 65 yrs	2.78	1.13–6.84	0.026	2.91	1.06–8.04	0.039
Severe asthma	12.29	5.30–28.54	< 0.001	8.91	3.69–21.49	< 0.001
**Severe asthma patients**						
Age of asthma onset	1.02	1.00–1.04	0.026	1.02	1.01–1.04	0.015
Exacerbations ≥ 3	4.45	1.84–10.74	0.001	4.88	1.98–12.03	0.001

OR: odds ratio. CI: confidence interval. The multivariate model is the final model after stepwise removal of covariates.

## Discussion

The present study found a high percentage of BQ in asthma patients, especially in those with severe asthma. In severe asthma patients, age of asthma onset and exacerbations were independently associated with the occurrence of BQ.

Though asthma and BQ are two different diseases, their coexistence has been demonstrated in many patients. In 2012, in a study applying HRCT Khadadah et al.^22^ reported the presence of BQ in 28.6% of persistent moderate asthma patients. In patients with persistent severe asthma, the presence of BQ is higher, ranging between 35%-80% according to the study^10,11,23–27^. In agreement with our results, in a large cohort of asthma patients Oguzulgen et al.^9^ recently reported that most asthma patients with BQ had severe persistent asthma (49.0%). In paediatric populations with severe asthma, approximately one-third of children had BQ, which was especially frequent in older children^28^. The differences observed between the studies may have been due to the study design or to the heterogeneity of the populations and comorbidities. Moreover, the widespread use of HRCT has increased the rate of identification of radiological bronchial dilatation and other airway abnormalities. Indeed, data from the US suggest that the number of annual HRCT procedures performed rose from 3 million in 1980 to 81.2 million in 2014^29^.

In the present study, asthmatic patients with bronchiectasis presented more frequent asthma exacerbations and worse asthma control. As we mentioned above, the coexistence of BQ and asthma has been observed in many patients, but few studies have been undertaken to investigate the relationship between the two diseases and the effect of BQ on exacerbations^30^. One such study, by Kang et al.^31^, found that BQ may be a risk factor for asthma exacerbation; these authors suggested that HRCT could be considered to search for concurrent BQ in uncontrolled asthma patients. In fact, BQ may be a terminal status in some patients with chronic airway inflammation, and early detection of the condition is recommended when patients present these features. Crimi et al.^32^ also advocate the use of HRCT to rule out BQ in patients with severe asthma and frequent exacerbations. A 5-year follow-up study reported a mortality rate of 20% in adults with BQ^33^. Bronchiectasis challenges asthma control and may aggravate the evolution of the condition.

Although asthma severity is an independent risk factor for BQ, the question of whether it is a cause of BQ remains unclear. In mild to moderate predominant BQ, asthma was the cause in 5.4% of cases^34^. Moreover, a Finnish study suggested that one-fourth of cases with BQ could be attributed to asthma^13^. The British guidelines also recommend considering asthma as the cause of BQ in the absence of any other etiological explanation^35^.

The present study found that, in the overall group, the factors independently associated with the occurrence of BQ were older age, asthma severity and frequent asthma exacerbations, while in patients with severe asthma the independently associated factors were age of asthma onset and frequent asthma exacerbations. In line with the results of the present study, Kang et al.^31^ found that annual incidence of asthma exacerbations, and emergency room visits due to asthma exacerbations were higher in patients with both asthma and BQ than in those with asthma alone, although the fact that the study population comprised mostly mild to moderate asthma patients may have influenced the results. In a cohort of moderate to severe asthma patients using the NOPES score (NOPES: nitric oxide, pneumonia, expectoration, and severity), Padilla-Galo et al. found these risk factors to be independently associated with BQ^36^.

We found that patients with asthma and BQ were more likely to present with sinusitis and nasal polyps. The association of sinusitis, nasal polyps and intolerance to aspirin, known as Samter's Triad Syndrome^37^, is a common phenomenon in patients with asthma and a relationship between asthma severity and nasal polyps has been reported^38^, but the pathology of BQ and its interaction with other nasal airway diseases requires further exploration. Nonetheless, in patients with BQ, nasal polyps and chronic sinusitis are quite frequent and are also characterized by early onset^39,40^. In fact, chronic sputum expectoration is common in patients with BQ, and mucus hypersecretion has been associated with rhinosinusitis and nasal polyps^41^. The present study also found that asthma patients with BQ were less atopic. In other research, atopy was significantly more prevalent in patients with asthma than in patients with chronic rhinosinusitis and nasal polyps^37^. Likewise, atopic dermatitis is less frequent in subjects with BQ than in those without (2% vs 11%, OR 0.188)^10^.

Bronchial wall thickening and bronchial dilatation are very common in severe asthma patients^42–44^, but few studies provide systematic quantification scores of these abnormalities in asthma patients. The present study describes the characteristics and severity of BQ, mainly finding its grade to be mild according to the criteria of Reiff^19^ and FACED^20^. Our results are consistent with the study by Padilla-Galo et al.^36^ with regard to bronchiectasis severity, since those authors recorded a mean FACED score of 1.45 pts, which also corresponds to mild grade disease. In contrast, they observed a mild predominant grade of bronchial wall thickening, whereas we found a notable proportion of patients with moderate predominant wall thickening. This difference may be attributed to the different degrees of asthma severity analysed in the two studies. In our study, 82% of patients presented cylindrical dilatation, similar to the rate reported by Padilla-Galo et al. (92.9%).

This study has several limitations. First, is a single-center study carried out at a clinic specialized in severe asthma. Larger studies should now be carried out in different settings to corroborate the data obtained. Secondly, the fact that the HRCT was carried out in some patients during the last three years may have introduced a bias. Third, in HRCT, low-dose volumetric acquisition protocol, with 1 mm cuts at 10 mm intervals in maximum inspiration was performed. In this sense, continuous 1 mm slices have a higher sensitivity than conventional HRCT images. Moreover, the bronchoarterial ratio could be influenced by aging. In this sense, the normal bronchoarterial ratio in up to 20% of healthy subjects older than 65 years overlaps with the ratio considered to represent bronchiectasis^8^. In our series, 53 patients (24%) were older than 65 years and in this group this radiological criterion may be present, which could alter the results. Finally, the characteristics of the study design do not allow us to differentiate whether the cause of the reduced FEV1 is asthma or the presence of BQ. However, we observed that patients with asthma and BQ have a lower FEV1 than those without BQ, which could mean that there is a synergistic effect between both conditions.

## Conclusions

Our results suggest that, in severe asthma patients, the presence of bronchiectasis is associated with age of asthma onset and the number of exacerbations. Our findings draw attention to the impact of BQ in asthma patients and should encourage clinical professionals to improve early detection measures and interventions for BQ in severe asthma patients, in order to improve patients’ quality of life and reduce the economic burden of this disease. Further studies are needed to confirm our results and to focus on the underlying mechanisms, for example via an assessment of the proinflammatory cytokines involved in this complex entity, and thus to be able to offer immunophenotype-based precision treatment.

## Data Availability

Not available in order to preserve participant confidentiality.

## Abbreviations

ACT: Asthma Control Test
ATS: American Thoracic Society
BQ: Bronchiectasis
COPD: Chronic Obstructive Pulmonary Disease
ERS: European Respiratory Society (ERS)
FEV1: Forced Expiratory Volume in 1 Second.
FVC: Forced Expiratory Volume in 1 Second.
GINA: Global Initiative for Asthma
HRCT: High-Resolution Computed Tomography
IQR: Interquartile Ranges
LAMA: Long-Acting Muscarinic Receptor Antagonists
NOPES: Nitric oxide, Pneumonia, Expectoration, Severity.
OR: Odds Ratio
SD: Standard deviations (SD)
SEPAR: Spanish Society of Pulmonology and Thoracic Surgery
VIF: Variance Inflation Factor
